# Apelin/APJ system: a novel promising target for anti-oxidative stress in stroke

**DOI:** 10.3389/fphar.2024.1352927

**Published:** 2025-01-15

**Authors:** Weilin Xu, Jun Yan, Zachary D. Travis, Cameron Lenahan, Liansheng Gao, Haijian Wu, Jingwei Zheng, Jianmin Zhang, Anwen Shao, Jun Yu

**Affiliations:** ^1^ Department of Neurosurgery, Second Affiliated Hospital, School of Medicine, Zhejiang University, Hangzhou, Zhejiang, China; ^2^ Key Laboratory of Precise Treatment and Clinical Translational Research of Neurological Diseases, Hangzhou, Zhejiang, China; ^3^ Department of Neurosurgery, Affiliated Tumor Hospital of Guangxi Medical University, Nanning, China; ^4^ Department of Medical Science Education, College of Health Sciences, Western University of Health Sciences, Pomona, CA, United States; ^5^ Burrell College of Osteopathic Medicine, New Mexico State University, Las Cruces, NM, United States

**Keywords:** apelin, APJ, stroke, oxidative stress, neuroprotection

## Abstract

The apelin/APJ system has garnered increasing attention in recent years. In this review, we comprehensively discuss the physiological and pathological mechanisms of the apelin/APJ system in stroke. The apelin/APJ system is widely expressed in the central nervous system (CNS). However, the distribution of the apelin/APJ system varies across different regions and subcellular organelles of the brain. Additionally, the neuroprotective effects of the apelin/APJ system have been reported to inhibit oxidative and nitrative stresses via various signaling pathways. Despite this, the clinical application of the apelin/APJ system remains distant, as apelin has numerous active forms and signaling pathways. The development of a range of drugs targeting the apelin/APJ system holds promise for treating stroke.

## Graphical Abstract

**Figure:**
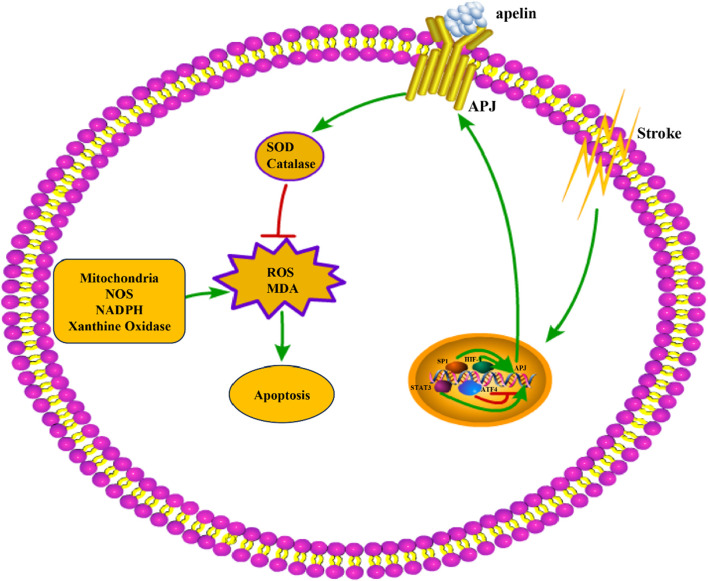
The Apelin/APJ system exerted neuroprotection after stroke via decreasing ROS levels and increasing antioxidant protein expression.

## 1 Introduction

Cerebrovascular diseases rank as the second leading cause of death, following heart diseases (Heron, 2007; Zhang et al., 2021a). Stroke is characterized by a sudden disturbance in blood flow, leading to mild or severe neurological dysfunction (The World Health Organization, 1988; World Health Organization, 2004). According to reports, 15 million people suffer from stroke annually, with 5.5 million succumbing to the disease (Banerjee et al., 2005). Stroke rates are increasing, particularly in developing countries, posing a significant societal burden (Zhang et al., 2021b; Shen et al., 2020).

Strokes can be classified as ischemic or hemorrhagic (Xu et al., 2018a; Wang et al., 2022; Xu and Zhong, 2013). They result from an interruption in the blood supply to brain tissues, leading to reduced oxygen and nutrients. Extensive research has been conducted on the pathological mechanisms and various therapeutic drugs for stroke, including studies on cellular apoptosis, oxidative stress, inflammation, brain edema, and cell death (Yi et al., 2013; Han et al., 2004). Currently, no drugs are specifically effective in treating stroke.

The apelin protein and its receptor APJ are extensively distributed in the brain, primarily located in oligodendrocytes and neurons (Gregory et al., 2004; Lee et al., 1993). Growing evidence suggests that apelin and its receptor APJ play crucial roles in protecting neural cells post-stroke (Bhalala et al., 2013; Tatemoto et al., 1998). Therefore, targeting the apelin/APJ system offers neuroprotection for stroke patients. This review aims to highlight the latest developments in the study of the apelin/APJ system’s functions and therapeutic potential in stroke patients.

## 2 Introduction of the apelin/APJ system

### 2.1 Apelin

The apelin gene expresses a 77-amino acid preproprotein, which is cleaved into several active peptides (Habata et al., 1999; Hosoya et al., 2000; Reaux et al., 2001). The full-length apelin, originating from bovine stomach extracts, consists of 36 amino acids. The presence of apelin-36 was further confirmed in bovine colostrum, and a 13-amino acid peptide (apelin-13) was also identified.

In the peptide, the N-terminus is modified by pyroglutamate, a post-translational modification that renders the protein resistant to enzymatic cleavage. Several potential proteolytic sites on apelin-36 suggest the existence of other endogenous apelin isoforms, such as apelin-19, apelin-17, apelin-16, and apelin-12, all of which can activate the APJ receptor (Kawamata et al., 2001; Tatemoto et al., 2001; Lee et al., 2005; Szokodi et al., 2002; Ronkainen et al., 2007). However, peptides containing fewer than 12 amino acids are inactive. In contrast to preproprotein, shorter forms of apelin exhibit higher binding affinity and greater activity, with pyroglutamated apelin-13 being the most potent.

Under hypoxic conditions, hypoxia-inducible factor-1 (HIF-1) upregulates apelin protein levels (Wang et al., 2006). During lactation, apelin synthesis is upregulated in breast tissue via upstream stimulatory factor-1 (Boucher et al., 2005). In fasting states, adipocyte apelin gene expression decreases, while refeeding stimulates its expression, possibly through changes in insulin and counter-regulatory hormone concentrations (Wei et al., 2005; Reaux-Le Goazigo et al., 2004). Furthermore, apelin expression in hypothalamic neurons is increased through the regulation of arginine vasopressin (Vickers et al., 2002). Post-translational processing of apelin is less understood, but angiotensin-converting enzyme 2 (ACE2) may be involved (O'Dowd et al., 1993). ACE2 efficiently hydrolyzes apelin-13 and apelin-36, although its physiological significance remains unknown.

### 2.2 The APJ receptor

The human APJ receptor, first reported by O'Dowd et al., is a G protein-coupled receptor with a 377-amino acid sequence located on chromosome 11 (Chng et al., 2013). In 1998, Tatemoto et al. identified apelin as the endogenous ligand of APJ (Habata et al., 1999). It wasn’t until 2013 that two groups independently discovered Elabela as another APJ ligand (Pauli et al., 2014; O'Carroll et al., 2006). The genetic regulation of APJ is complex, with a TATA-less promoter region playing a role in APJ gene expression. Physiological stimuli such as stress, salt loading, and water deprivation induce APJ synthesis (Pugh and Tjian, 1991). Several genes with TATA-less promoters are activated by Sp1 (Japp and Newby, 2008), which is also crucial for APJ promoter activation. Other factors influencing promoter activity include estrogen, CCAAT/enhancer-binding protein, and glucocorticoids (Pugh and Tjian, 1991).

### 2.3 Distribution of the apelin/APJ system in the CNS

**FIGURE 1:**
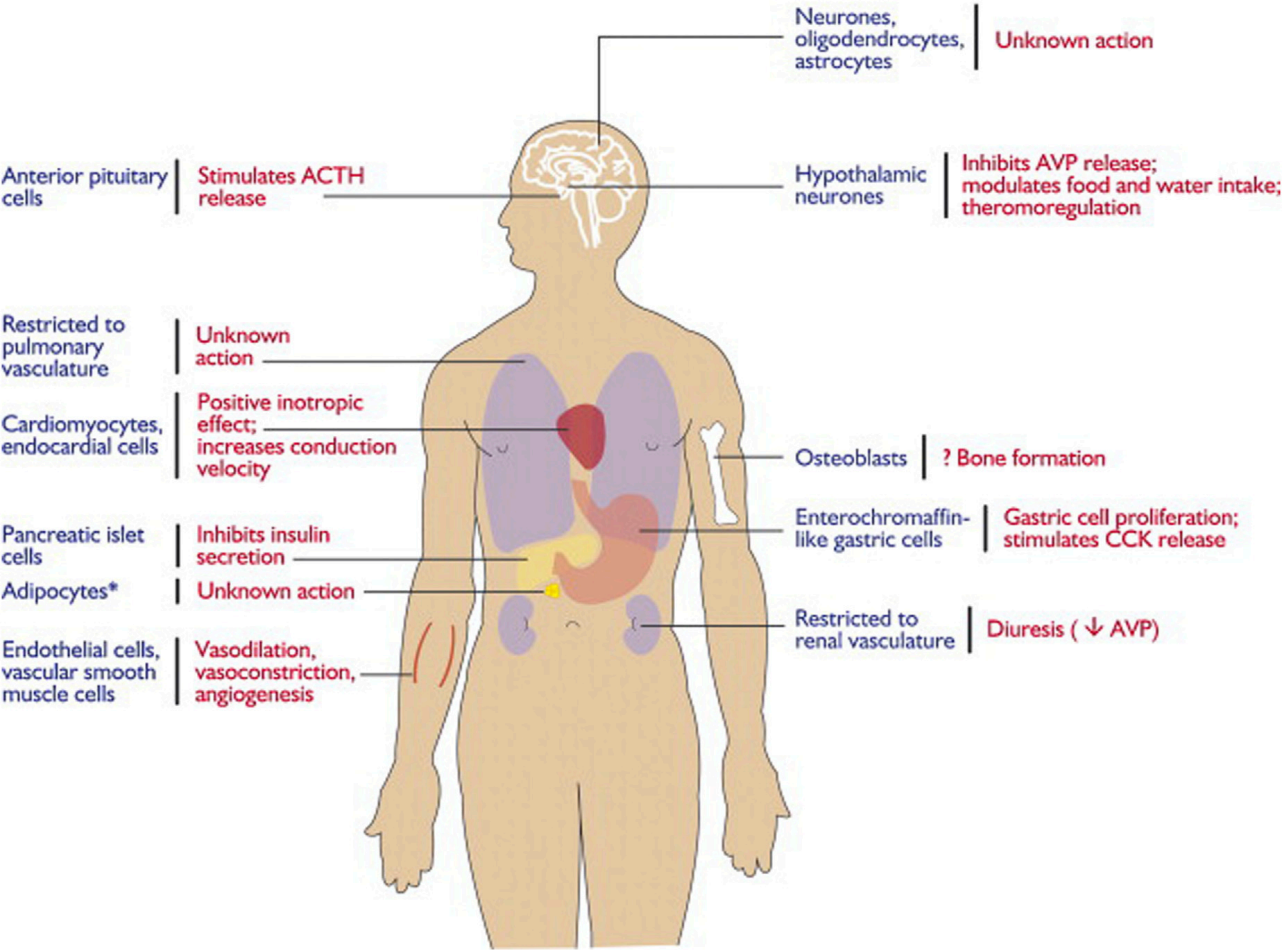
Expression and physiological functions of the apelin–APJ system. Reproduced with permission from ref. (Chng et al., 2013).

The apelin/APJ system is extensively distributed in the CNS and other tissues (Lee et al., 2000) (Figure 1). In detail, the apelin/APJ system is expressed in neurons of the cerebral cortex, pituitary gland cells, hippocampus, hypothalamus, T-lymphocytes, and pancreatic islet cells. Apelin expression levels are associated with APJ expression.

**FIGURE 2:**
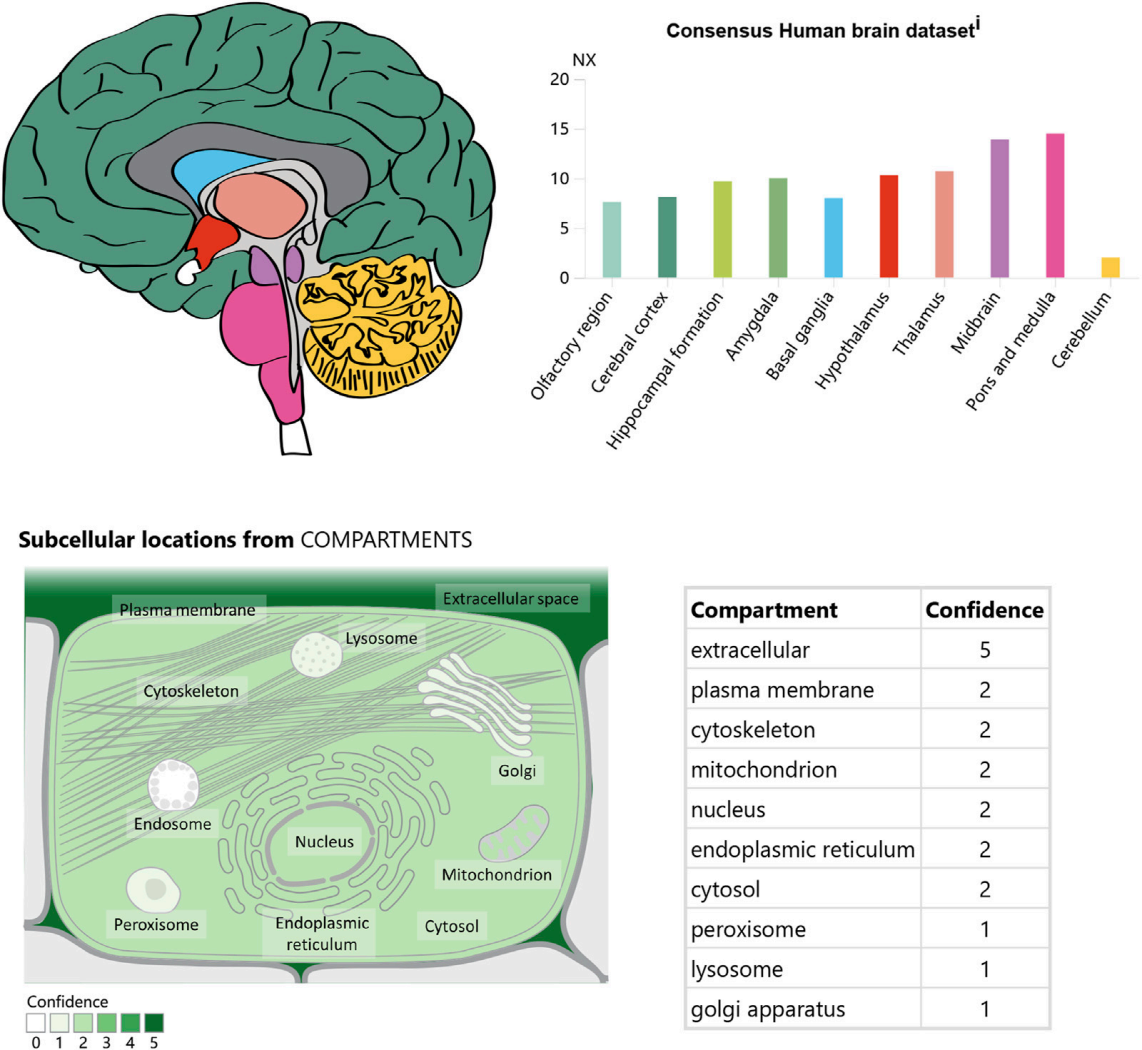
The expression and location of Apelin in the brain. Referenced from the human protein atlas: [Apelin-APJ system] (https://www.proteinatlas.org/ENSG00000134817 - APLNR/brain).

In the CNS, apelin is expressed both centrally and peripherally. Northern blot analysis detected apelin mRNA in the CNS of rats (O'Carroll et al., 2000; Medhurst et al., 2003). The highest mRNA expression of apelin was observed in the cerebral cortex, hippocampus, pineal gland, spinal cord, and olfactory tubercle of rats (O'Carroll et al., 2000; Reaux et al., 2002) (Figure 2). Immunocytochemical techniques identified apelin-LI in other neural cells, including neurons of the hypothalamus, pons, and medulla oblongata (Kawamata et al., 2001; Kleinz et al., 2005). In humans, apelin expression was first reported in the hippocampus, thalamus, basal ganglion, hypothalamus, frontal cortex, and basal forebrain (O'Carroll et al., 2000). Another study localized apelin mRNA in the corpus callosum, spinal cord, substantia nigra, amygdala, and pituitary (Reaux et al., 2002). Apelin-LI is expressed in the endoplasmic reticulum, secretory vesicles, and Golgi complex of endothelial cells, but not in cell organelles of inducible endothelial peptide secretion (Lee et al., 2004).

**FIGURE 3:**
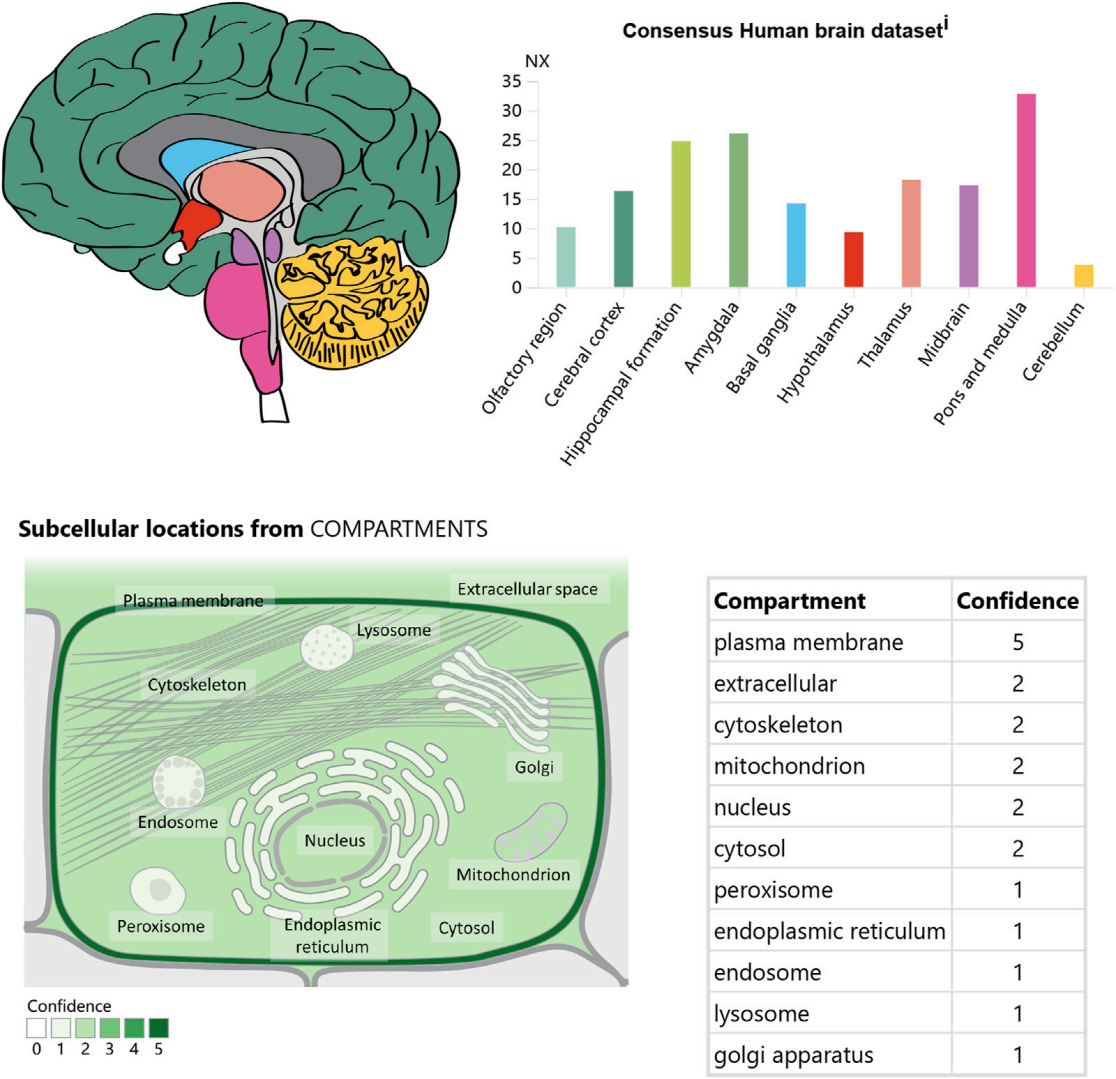
The expression and location of APJ in the brain. Referenced from the human protein atlas: [APJ System] (https://www.proteinatlas.org/ENSG00000171388 -APLN/brain).

In the CNS, APJ can be detected in the hippocampus, cerebral cortex, pituitary gland, and hypothalamus, with the highest levels in the hypothalamus (O'Carroll et al., 2000; Medhurst et al., 2003) (Figure 3). Immunocytochemical detection showed that apelin receptors are also expressed in the nuclei of several neuron types, including hypothalamic and thalamic nuclei (Reaux et al., 2002). APJ expressed in human embryonic kidney cells shares similar characteristics with that expressed in rat hypothalamic and cerebellar nuclei (Choe et al., 2000).

Studies on cells expressing APJ in the CNS revealed its presence in neurons, astrocytes, and oligodendrocytes, but not in macrophages or microglia. Immunocytochemical analysis using an antibody against the apelin receptor confirmed its distribution in the human brain (Masri et al., 2006). APJ-LI was detected only in human pyramidal and cerebellar neurons in culture.

### 2.4 Biochemistry: intracellular signaling mechanisms

Studies have shown that apelin inhibits the production of forskolin-stimulated cyclic AMP, suggesting that APJ is coupled with inhibitory G proteins (Gi) (Habata et al., 1999). Additionally, apelin can activate extracellular-regulated kinases (ERKs) in a Ras-independent manner (Masri et al., 2002) and activate p70S6 kinase in an ERK- and Akt-dependent manner (Neves et al., 2002). Pertussis toxin inhibits these signaling cascades, indicating that they are supported by APJ-Gi coupling. However, pertussis toxin does not completely suppress apelin’s inotropic effect (Ronkainen et al., 2007) but can activate phospholipase C and protein kinase C, which are activated by Gq proteins (Zhou et al., 2003). Therefore, it is possible that APJ receptors couple with both Gq and Gi proteins. Phosphatidylinositol-4,5-bisphosphate (PIP2) can be hydrolyzed by phospholipase C to produce inositol-1,4,5-triphosphate (IP3) (Zhou et al., 2003), demonstrating that apelin can increase intracellular Ca2+ concentrations (Masri et al., 2006). Notably, several apelin-mediated signaling cascades are reduced in sensitization following activation (Masri et al., 2002), likely due to the internal positioning of APJ receptors (Eggena et al., 1979). Different sizes of apelin fragments lead to different durations of receptor internalization, correlated with varying patterns of desensitization (Masri et al., 2002; Eggena et al., 1979). Finally, APJ receptors are also localized to the nucleus (Choe et al., 2000), suggesting they can regulate transcription in addition to activating intracellular cascades (Tang et al., 2021).

## 3 Oxidative stress in the CNS

### 3.1 Characteristics of oxidative stress in the CNS

Oxidative stress causes significant damage to organs under ischemic conditions, including the heart, liver, kidneys, and especially the brain (Andrianova et al., 2020; Yang et al., 2016; Ikeda and Miyahara, 2003; Frank, 2006). Anatomical, physiological, and functional factors make the brain particularly susceptible to oxidative injury. Human brains consume 20% of the body’s oxygen due to their high metabolic rate, although they account for only 2% of body weight. This higher oxygen availability results in increased ROS production (Gu et al., 2011). The brain’s dependence on glymphatic waste disposal, modest antioxidant defenses, excitotoxic and auto-oxidizable neurotransmitters, polyunsaturated fatty acids prone to peroxidation, limited regenerative capacity, redox-active metal burden, and calcium load make it sensitive to ROS.

**FIGURE 4:**
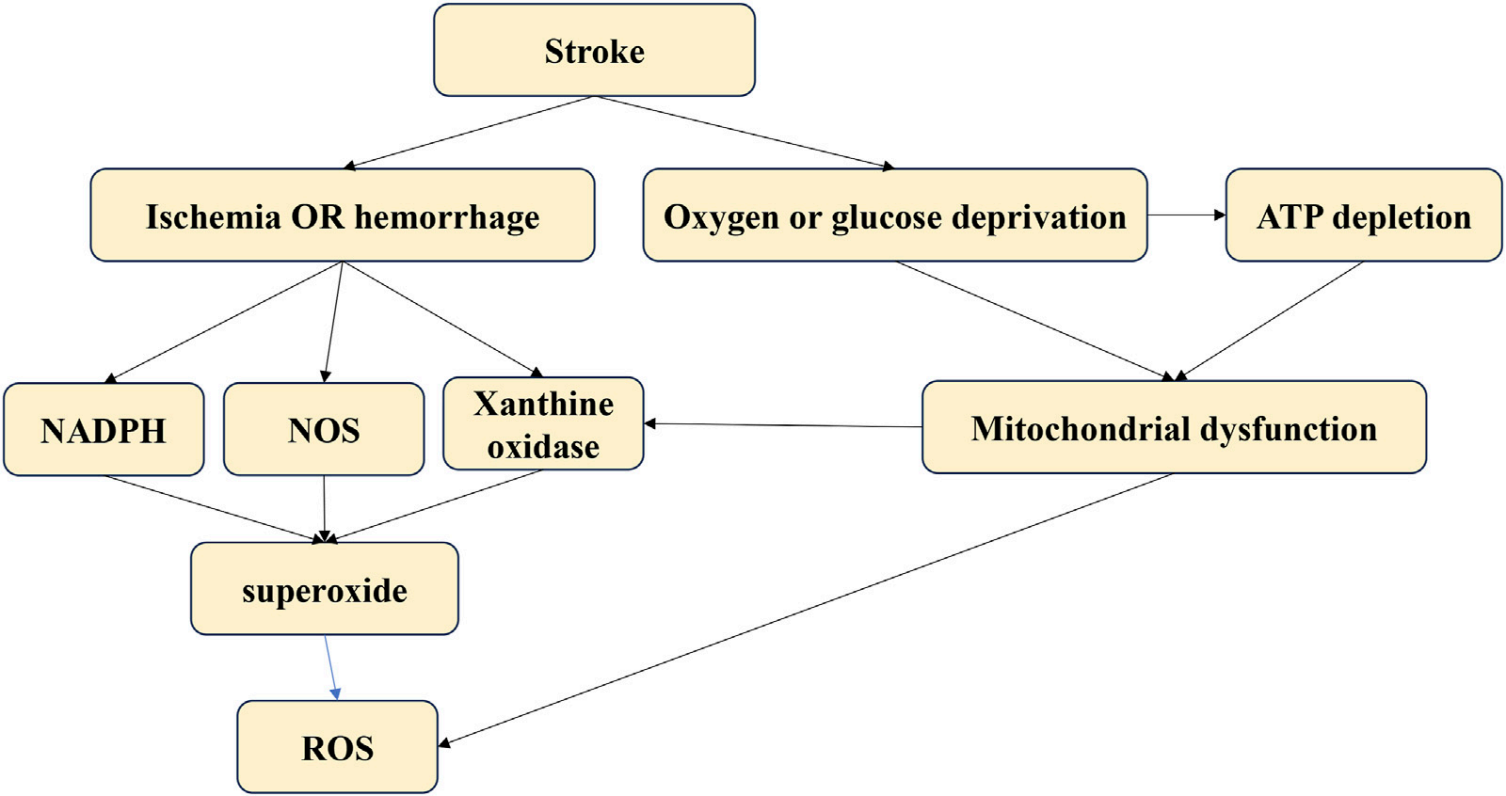
The different sources of oxidative stress.

Oxidative stress-related neurofunctional damage may result from various cellular pathophysiological processes within neural cells. The oxidative stress vulnerability of neurons differs biochemically. Psychological stress may compromise antioxidant enzyme function by disrupting the oxidant-antioxidant balance in the brain, depleting glutathione and increasing oxidative stress. When glutamate toxicity occurs simultaneously with mitochondrial dysfunction, oxidative stress, and calcium overload, it leads to brain damage, impaired neural cell communication, and ultimately neuro-dysfunction. Controlling ROS levels, either by quenching pro-oxidants or enhancing antioxidant defense, is crucial for the CNS. This review provides a biologically plausible explanation of how oxidative damage might contribute to psychiatric symptoms [Figure 4].

### 3.2 Signal pathways of ROS in the brain

The mechanisms by which ROS induce brain injuries remain unclear. However, ROS have been demonstrated to cause various cellular pathologies, including blood-brain barrier disruption, neuronal apoptosis, and neuroinflammation (Makino et al., 1996). Evidence suggests that N-methyl-D-aspartate receptor glutamate toxicity and glucocorticoid receptor signaling are involved (Okamoto et al., 1999; Tanaka et al., 1999; Albrecht et al., 2010; Nguyen et al., 2011; Sorce and Krause, 2009). Neurodegenerative diseases such as cerebrovascular disorders, Parkinson’s disease, and Alzheimer’s disease have been linked to increased brain oxidative damage (Kamata et al., 2005).

Oxidative stress is believed to damage macromolecules by activating particular signaling pathways in cells, altering gene expression, and leading to cell death (Finkel, 2000; Meng et al., 2002; Hu and Wieloch, 1994). The mechanisms linking oxidative stress signals to cellular responses remain unclear, but several proteins play important roles, including mitogen-activated protein kinases (MAPKs: JNK, ERK1/2, ERK5, p38MAPK) (Kuan et al., 2003; Suzaki et al., 2002; Dunah et al., 2002), Sp1 (Susin et al., 1999), oxidoreductase apoptosis-inducing factor (AIF) (Vahsen et al., 2004; Chalovich et al., 2006), transcription factors such as cAMP response element-binding protein (CREB) (Zaman et al., 1999; Van Der Heide et al., 2004), and Forkhead (FOXO) (Brunet et al., 2004; Essers et al., 2004; Orellana-Urzúa et al., 2020).

## 4 The functions of the apelin/APJ system in stroke

### 4.1 Pathological mechanisms of stroke

Strokes result from an interrupted blood supply to brain tissues, leading to reduced oxygen and nutrients. The lack of blood supply causes a rapid increase in ROS production immediately after an acute stroke. These ROS can damage neural cells, leading to further pathological processes such as neuroinflammation, autophagy, neuro-apoptosis, and blood-brain barrier disruption (Rodrigo et al., 2013). The main sources of ROS after a stroke include mitochondria, nitric oxide synthases, NADPH oxidase, and xanthine oxidase (Wu et al., 2017). Targeting oxidative stress post-stroke is critical to preventing oxidative damage and subsequent pathological processes in stroke patients.

### 4.2 Expression of apelin/APJ after stroke

Apelin and APJ expression levels vary at different time points during a stroke (Lv et al., 2013). In one of my previous studies, we found that the endogenous level of apelin-13 increased at 12 h and peaked at 24 h after SAH, while the levels of APJ started to increase at 6 h and peaked at 24 h after SAH (Xu et al., 2019). Various transcription factors, such as ATF4, Sp1, STAT3, and HIF-1α, play crucial roles in regulating the apelin/APJ system (Pugh and Tjian, 1991; He et al., 2015; Han et al., 2008; Zhang et al., 2009; Yeh et al., 2011). Cerebral ischemia results in oxygen and glucose deprivation, which is associated with abnormal apelin/APJ signaling (Zhang et al., 2009; Yeh et al., 2011). HIF-1α and Sp1 induce the expression of apelin/APJ after a stroke (Pugh and Tjian, 1991; He et al., 2015; Han et al., 2008). Under ischemic conditions, HIF-1α translocates to the nucleus and activates the transcription and expression of apelin and APJ proteins (Wang et al., 2006; Han et al., 2008). Apelin and APJ expression is induced in neurons during the early stages of ischemia by increased Sp1 via HIF-1α (Woo et al., 2012; Fan et al., 2017). Reperfusion, however, results in the downregulation of the apelin/APJ system. In mice subjected to chronic normobaric hypoxia, APJ expression in the hippocampus is significantly reduced, and apelin-13 can reverse this reduction (Sheng et al., 2012). The apelin/APJ system is also affected by ER stress, inflammation, and oxidative stress (Mei et al., 2015; Morimoto et al., 2007). For example, cerebral I/R injury is mediated by the ER stress response, which is activated during reperfusion but not ischemia (Nakka et al., 2010; Xin et al., 2014; Jeong et al., 2014). Reperfusion may induce apelin expression due to ER stress regulation by ATF4 via the p38 MAPK pathway (Liu et al., 2018). Apelin-12 has been shown to inhibit the JNK and p38MAPK signaling pathways, leading to cell apoptosis in MCAO-induced ischemic mice (Arani Hessari et al., 2022).

Due to the crucial roles of the apelin/APJ system in stroke, targeting it could provide novel treatments for stroke.

### 4.3 Modulation of oxidative stress by the apelin/APJ system after stroke

The apelin/APJ system is widely studied for its neuroprotective properties, including anti-neuroinflammation, anti-apoptosis, and antioxidative stress. Several studies have reported the neuroprotective effects of the apelin/APJ system in the CNS. However, research on stroke has largely focused on its anti-apoptosis and anti-inflammatory effects, overlooking its antioxidative stress effects (Gholamzadeh et al., 2021; Xu et al., 2018b; Xu et al., 2019). This study focuses solely on the antioxidative stress effects of the apelin/APJ system, providing guidance for future research.

The apelin/APJ system inhibits oxidative and nitrative stresses, producing neuroprotective effects. Our previous study showed that the apelin/APJ system exerts significant antioxidative effects by suppressing endoplasmic reticulum stress-associated oxidative stress via AMPK/TXNIP/NLRP3 signaling pathways (Wu et al., 2015). It also increases superoxide dismutase activity and decreases malondialdehyde (MDA) levels to reduce oxidative stress induced by I/R injury (Duan et al., 2019). Apelin-13 has been shown to significantly decrease ROS and MDA levels while increasing antioxidant protein expression in a dose-dependent manner via the AMPK/GSK-3β/Nrf2 pathway (Khoshnam et al., 2017). The apelin/APJ system may protect cells from oxidative stress-induced death by decreasing ROS production and promoting ROS clearance.

Nitric oxide (NO) exhibits different effects in ischemic stroke: it is neuroprotective when produced by endothelial NOS (eNOS) but mediates oxidative/nitrosative injuries when generated by neuronal NOS (nNOS) (Iadecola et al., 1997; Salcedo et al., 2007). Similarly, apelin has dual effects on vascular function. Activation of the apelin/APJ axis induces peripheral arterial relaxation in a NO- and endothelium-dependent manner (Japp et al., 2008; Modgil et al., 2013). However, in male rats, the apelin/APJ system inhibits NO-induced cerebral artery relaxation by blocking calcium-activated K (BKCa) channels via the PI3K/Akt pathway (Mughal et al., 2018; McKinnie et al., 2017). Further research is needed to determine how apelin affects oxidative/nitrosative stress in ischemic stroke.

## 5 Potential targets of the apelin/APJ system

As indicated above, the apelin/APJ system plays a major role in the occurrence and development of several diseases, including strokes. Therefore, targeting the apelin/APJ system can be a promising approach to treating neurological diseases (Brame et al., 2015). Recent studies have identified small molecule agonists and antagonists targeting the apelin/APJ system. For example, an antidiuretic hormone-inducing non-peptide agonist E339-3D6 has been reported to induced vasorelaxation of rat aorta precontracted with noradrenaline and potently inhibited systemic vasopressin release by activating with apelin receptor, which can be a potential target to allow development of a new generation of vasodilator and aquaretic agents (Murza et al., 2014). [20040517]. Additionally, ML233 selectively inhibits AT1 receptors, inducing vasoconstriction via phospholipase C by binding to APJ (Gerbier et al., 2017). These molecules can decrease renin levels generated via the cAMP pathway (Murza et al., 2012). Research on antagonists of APJ receptors is also progressing rapidly. ML221 was the first such antagonist to be developed (Jia et al., 2012); another antagonist, ALX40-4C, has also been identified. The ALX40-4C receptor antagonist, consisting of nine arginine residues, is effective for both APJ and CXCR4 receptors and inhibits intracellular calcium mobilization and receptor internalization in response to ligands, and it can be the potential utility for further elucidation of HIV-1 neuropathogenesis and therapy of HIV-1-induced encephalopathy (Jia et al., 2012). PMID: 12890632 Additionally, some apelin-13-based molecules showed more potent and stable analogs targeted at APJ (Juhl et al., 2016). Cao et al. showed that apelin analogs directly reduced blood pressure by activating the Akt-eNOS/NO pathway. Drugs targeting apelin might help treat inflammation-related diseases associated with oxidative stress. Puerarin has been shown to reduce apelin expression and protect against renal hypertension (Juhl et al., 2016; Centers for Disease Control and Prevention, 2001), suggesting its potential in treating oxidative stress-linked blood pressure. To conclude, Apelin/APJ-targeting drugs contribute to pharmacological research and understanding the mechanism of oxidative stress-mediated diseases.

## 6 Conclusion

The apelin/APJ system is extensively distributed in the brain and plays vital roles in regulating neurological diseases, including stroke. It shows significant neuroprotective effects by suppressing oxidative and nitrative stresses via different signaling pathways. Recent years have seen the discovery of potential drugs targeting APJ and apelin (E339-3D6, ML233, ML221, and ALX40-4C), and pharmacological interactions with Apelin/APJ have become reliable tools for exploring this system’s role in oxidative stress-mediated diseases.

Further in-depth studies on the physiological and pathological effects of the apelin/APJ system and its potential mechanisms will greatly aid clinical prevention and intervention in strokes. The development of drugs targeting the apelin/APJ system will benefit patients and alleviate the pressures on families and society.

This study extensively shows the apelin/APJ system and its antioxidative roles in stroke. However, some limitations should be addressed: 1. This study focuses solely on the antioxidative effects of the apelin/APJ system in stroke, overlooking other physiological roles such as anti-apoptosis and anti-neuroinflammation. Application of this results should be more carefully. 2. Although some clinical trials have been registered (Num. ChiCTR2200060945, ChiCTR2100054712, ChiCTR-OOC-15006043, ChiCTR-ODT-13004019), clinical application of apelin/APJ has not yet been reported. The clinical values of apelin/APJ system should be further explored in future studies. 3. Currently, limited studies focus on the antioxidative effects of the apelin/APJ system in stroke, which remains largely unexplored. Future studies of the apelin/APJ system should include different cellular signaling pathways of oxidative stress, various sources of ROS, and different clinical drugs that can be further explored. 4. The connection of different signaling pathways mediated by apelin/APJ system should be further explored, which can greatly increase the readability and application of apelin/APJ system.

## Abbreviations

CNS, central nervous system; WHO, World Health Organization; HIF-1, hypoxia-inducible factor-1; ACE2, angiotensin-converting enzyme 2; ERKs, extracellular-regulated kinases; PIP2, phosphatidylinositol-4,5-bisphosphate; IP3, inositol-1,4,5-triphosphate; DG, dentate gyrus; ROS, radical oxidative stress; AIF, apoptosis-inducing factor; CREB, cAMP response element-binding protein; ATF4, activating transcription factor 4; STAT3, signal transducer and activator of transcription 3; HIF-1α, hypoxia-inducible factor 1 alpha; MAPK, mitogen-activated protein kinase; JNK, C-Jun N-terminal kinase; MDA, malondialdehyde; SOD, superoxide dismutase; NO, nitric oxide; BKCa, blocking calcium-activated K.
